# Multivariate analysis of biologging data reveals the environmental determinants of diving behaviour in a marine reptile

**DOI:** 10.1098/rsos.211860

**Published:** 2022-08-10

**Authors:** Jenna L. Hounslow, Sabrina Fossette, Evan E. Byrnes, Scott D. Whiting, Renae N. Lambourne, Nicola J. Armstrong, Anton D. Tucker, Anthony R. Richardson, Adrian C. Gleiss

**Affiliations:** ^1^ Centre for Sustainable Aquatic Ecosystems, Harry Butler Institute, Murdoch University, Western Australia, Australia; ^2^ Environmental and Conservation Science, Murdoch University, Western Australia, Australia; ^3^ Biodiversity and Conservation Science, Department of Biodiversity, Conservation and Attractions, Kensington, Western Australia, Australia; ^4^ Parks and Wildlife Service, West Kimberley District, Department of Biodiversity, Conservation and Attractions, Broome, Western Australia, Australia; ^5^ Faculty of Science, Simon Fraser University, British Columbia, Canada; ^6^ School of Electrical Engineering, Computing and Mathematical Sciences, Curtin University, Bentley, Western Australia, Australia

**Keywords:** sea turtle, biologging, multi-sensor data, diving behaviour, dive type

## Abstract

Diving behaviour of ‘surfacers' such as sea snakes, cetaceans and turtles is complex and multi-dimensional, thus may be better captured by multi-sensor biologging data. However, analysing these large multi-faceted datasets remains challenging, though a high priority. We used high-resolution multi-sensor biologging data to provide the first detailed description of the environmental influences on flatback turtle (*Natator depressus*) diving behaviour, during its foraging life-history stage. We developed an analytical method to investigate seasonal, diel and tidal effects on diving behaviour for 24 adult flatback turtles tagged with biologgers. We extracted 16 dive variables associated with three-dimensional and kinematic characteristics for 4128 dives. *K*-means and hierarchical cluster analyses failed to identify distinct dive types. Instead, principal component analysis objectively condensed the dive variables, removing collinearity and highlighting the main features of diving behaviour. Generalized additive mixed models of the main principal components identified significant seasonal, diel and tidal effects on flatback turtle diving behaviour. Flatback turtles altered their diving behaviour in response to extreme tidal and water temperature ranges, displaying thermoregulation and predator avoidance strategies while likely optimizing foraging in this challenging environment. This study demonstrates an alternative statistical technique for objectively interpreting diving behaviour from multivariate collinear data derived from biologgers.

## 1. Introduction

The diving behaviour of air-breathing aquatic animals is primarily governed by access to two spatially disparate, yet vital resources—food and oxygen [1–3]. Understanding movement between these two resources via the study of diving behaviour is therefore central to understanding population-level processes in aquatic air-breathers (*sensu* [4]). Since *in situ* behavioural observations are logistically challenging for extended periods underwater, animal-attached tags or biologgers have revolutionized the study of diving behaviour for free-ranging aquatic animals [5]. The earliest biologgers were developed to record time and depth data, quantifying the depth and duration of dives for pinnipeds (e.g. Weddell Seals, *Leptonychotes weddellii*) and seabirds (e.g. emperor penguins, *Aptenodytes forsteri*) [6,7]. Time-depth data have taught us about the timing, frequency and location of ecologically important behaviours, such as foraging and resting, in relation to environmental conditions for a range of diving animals [8–10]. In particular, classification of dive types based on the geometric shape of dive profiles has enabled behaviours to be inferred and quantified at the scale of individual dives [11–14]. For example, seabirds and pinnipeds almost exclusively perform U-shaped dives that have generally been associated with foraging, while V-shaped dives have been assumed to represent travelling or searching [15].

Since the 1980s, biologgers have been increasingly deployed to a broader range of marine taxa [16,17]. Techniques used to interpret diving behaviour from time-depth data were originally developed for divers. Unlike ‘divers' who predominantly remain at or above the surface, ‘surfacers' such as sea snakes, cetaceans and sea turtles [18] remain at-sea and submerged for extended durations. While divers almost exclusively dive to search for and capture food, surfacers can perform a diverse range of behaviours while diving (e.g. foraging, searching, resting, socializing, mating, travelling) and indeed within the same dive [19]. For example, sea turtles both rest and forage during U-shaped dives [20–24]. Hence, interpreting diving behaviour for these animals is more complicated, and this complexity may be better represented by multi-dimensional data.

Two-dimensional data have previously been argued to be a simplistic, often subjective representation of multi-dimensional diving behaviours [25], and as a result may not always be sufficiently descriptive [26]. Beyond time and depth, the interpretation of diving behaviour can be enhanced by other parameters such as swim speed, body angle, stroke frequency and path heading [27–30]. This requirement to quantify additional aspects of diving behaviour has driven the increasing diversification of sensors available in biologgers [31–33]. Integrating multiple sensors (e.g. animal-borne video, accelerometer, magnetometer) into one device is now prevalent in the study of animal behaviour, physiology and ecology [34,35]. High-resolution motion sensors are especially useful for inferring behaviour in the absence of direct observation, based on the fine-scale kinematics that characterize patterns in locomotion and body posture [36,37]. For example, simultaneous measurements of swim speed, compass heading, depth and video from northern elephant seals (*Mirounga angustirostris*) revealed energy efficient diving strategies achieved through prolonged gliding during dive descents [38,39]. More recently, accelerometry has revealed benthic resting by southern elephant seals (*M. leonina*), highlighting that this resting behaviour was previously either mislabelled as swimming or not detected at all when relying on time-depth data alone [40]. For sea turtles, aside from animal-borne video data [24], detailed descriptions of diving behaviour and associated physical activity have been obtained from both measurements of acceleration and angular velocity [41–46], as well as three-dimensional reconstruction of underwater travel paths via dead-reckoning [47,48].

Technological advancements have given us the ability to collect new parameters of diving behaviour, yet it remains challenging to analyse these ‘big' multi-faceted datasets [49,50]. Indeed, refining our analysis of biologging data in general has been flagged as a high priority [35]. In addition, considering the evolution of techniques used to analyse diving data has been highlighted as a valuable lesson in itself [15]. In the view of the rapidly increasing uptake of biologging technologies, part of this evolution and refinery includes finding methods to incorporate multiple streams of high-resolution data into diving behaviour analysis for surfacers.

The flatback turtle (*Natator depressus*) is likely no exception to the complex diving behaviours exhibited by surfacers; however, unlike other species there is limited data describing it [51]. To date, flatback turtle diving behaviour has only been investigated from time-depth data [52–56]. Although globally listed as ‘data deficient' [57], the flatback turtle is relatively well studied during hatchling, nesting, inter-nesting and post-nesting migratory life-stages, but the foraging stage remains largely unstudied [58,59]. This is mainly because until recently, access to areas used by foraging individuals at-sea has not been possible [55,60]. The discovery of a year-round coastal aggregation of flatback turtles in the Kimberley region of northern Western Australia now offers reliable access to study the foraging life-history stage [61].

Flatback turtles are endemic to continental shelf waters of northern Australia [62], therefore their relatively limited range makes them particularly vulnerable to threats, such as climate change [63,64] and offshore oil and gas industrial development [65]. Consequently, it is important not only to have a good understanding of flatback turtle diving behaviour, but also its environmental drivers, to inform future conservation management. Environmental conditions directly and indirectly impact the behaviour of aquatic species over varying spatiotemporal scales, such as daily movements, yearly migrations and potential range shifts [35,66]. For instance, sea turtles are susceptible to both acute (i.e. seasonal) and chronic (i.e. climate change) changes in water temperature, which directly affect physiological processes and associated diving performance [18,67,68]. Tides, which alter depth and current in a given place, can be another important factor influencing the behaviour of coastal species [69], especially for the flatback turtle given its distribution across the highly tidal waters of northern Australia.

The overall aim of this study is to use high-resolution multi-sensor biologging data to provide the first detailed description of the environmental variables that influence the diving behaviour of flatback turtles during their foraging life-history stage. Specifically, our first objective was to develop an analytical method to interpret the diving behaviour of surfacers from kinematic and three-dimensional variables derived from multi-sensor data. In doing so, our second objective was to investigate the seasonal, diel, and tidal effects on flatback turtle diving behaviour.

## 2. Material and methods

### 2.1. Study site

Yawuru Nagulagun Roebuck Bay (−18.05°, 122.15°) is a 360 km^2^ embayment of the Indian Ocean near Broome, northern Western Australia, experiencing a semi-arid monsoonal climate. The bay is mostly shallow (less than 20 m), characterized by semi-diurnal tides with a large tidal range (approx. 10 m), featuring extensive intertidal mud and sand flats and mangroves surrounding much of the bay [70,71].

### 2.2. Field protocol

Fieldwork was completed between 2018 and 2020 over Austral winter and summer seasons. Flatback turtles (*n* = 28) were captured by hand from a 7 m research vessel using either a hoop net or the rodeo technique [72] during haphazard surveys of the study site. Once captured, turtles were brought on-board the vessel, measured (Curved Carapace Length, CCL), weighed, sexed, and given unique identification markers (titanium flipper tag and intra-muscular Passive Integrated Transponder (PIT) tag). Turtles were instrumented with a CATS (Customized Animal Tracking Solutions, QLD, Australia) multi-sensor tag; either a Camera (dimensions 250 × 140 × 50 mm and weight 758 g) or Diary (190 × 110 × 45 mm and weight 622 g), with both tag types similarly equipped with multiple high-resolution sensors. Refer to electronic supplementary material, table S2 for details. The tag was attached to the carapace along the midline on the second vertebral scute, using either rubber suction cups as per Hounslow *et al*. [61] (electronic supplementary material, figure S1A), or a custom-made self-detaching harness (electronic supplementary material, figure S1B). The harness was made of polyester-webbing and Velcro^®^ and included a plastic-neoprene padded baseplate, to which the Diary was securely attached using cable ties. The harness was fitted to the turtle as per Sperling and Guinea [73]. Both types of multi-sensor tag (Camera and Diary) were programmed to record data collected by tri-axial accelerometer, magnetometer and gyroscope (20–50 Hz) and pressure and temperature (10 Hz) sensors. The tags' Global Positioning System (GPS) was programmed to record duty-cycled location data (Coordinate Reference System WGS 84), for alternate hours during the day and every third hour overnight to reduce power demand and maximize deployment durations. Both tags feature an internal GPS antenna, therefore a depth trigger was set to collect GPS data only when depth was less than 1 m, when the turtle was at or near to the surface. Tagged turtles were released at the capture location after less than 30 min on board the vessel. A galvanic timed release (GTR, Ocean Appliances Australia) mechanism released the tag from the turtle after a pre-determined time (24 h to 7 days). The positively buoyant tag package, combined with a satellite SPOT-258 (Wildlife Computers) and VHF tag (Advanced Telemetry Systems), transmitted location data upon floating to the surface. Tag packages were recovered by VHF telemetry and ARGOS satellite location (www.argos-system.org/).

### 2.3. Data pre-processing and extraction of dive variables

The first 2 h of data were removed from each individual record to ensure effects of capture were not included in analysis [74]. Multi-sensor data were pre-processed using Igor Pro (v. 8; Wavemetrics Inc; Lake Oswego, USA) and the *R* statistical environment (v. 4.0.3) [75]. A set of 16 descriptive dive variables were extracted from the multi-sensor data to characterize dives (summarized in table 1). Zero offset corrections (ZOC) were first applied to the entire depth record, based on depth when the turtle was at the surface. Depth was resampled to 1 Hz using the *resample* function in Igor. To distinguish single dives as measurement units from each turtle's depth record, we used the *R* package *diveMove* (v. 1.5.2) [76]. A minimum depth threshold of 1 m (separating diving from surface behaviours) and duration greater than 30 s were used to define a dive. Dives and their phases (descent, bottom, ascent and post-dive surface interval) were identified using the *createTDR* and *calibrateDepth* functions. Within each dive, we used the *diveMove* function *getStats* to calculate summary statistics related to depth and the duration of each dive phase. For full implementation of *diveMove*, see electronic supplementary material, section S1.1.

**Table 1.** Descriptive dive variables calculated from high-resolution (10–50 Hz) multi-sensor data collected from flatback turtles (*N. depressus*) at Yawuru Nagulagun Roebuck Bay, Western Australia. Variables were calculated for each dive for all individuals and combined into a data matrix.

metric	variable	description	unit
duration	dur.desc	duration of each dive phase including the post dive surface interval (pdsi)	minutes (min)
	dur.bott		
	dur.asc		
	dur.pdsi		
depth	bottdist	sum change in depth during bottom phase	metre (m)
	bottdep	mean depth of bottom phase	
	maxdep	maximum depth of bottom phase	
activity	odba.desc	mean ODBA for each dive phase	*g*
	odba.bott		
	odba.asc		
body angle	angle.desc	mean pitch for each dive phase	degrees (°)
	angle.bott		
	angle.asc		
tortuosity	circ.var.desc	circular variance of heading for each dive phase	*V*
	circ.var.bott		
	circ.var.asc		

^a^Dives and dive phases (descent, bottom, ascent and post dive surface interval) identified using *diveMove* v. 1.5.2 in R.

Additional variables related to locomotory activity, body angle and tortuosity were calculated for each dive phase, to further characterize multi-dimensional aspects of dives (see expanded definitions below and table 1). We used a single metric, overall dynamic body acceleration (ODBA) as a proxy for locomotory activity and associated energy expenditure [77]. Acceleration data, measured at 20–50 Hz in three orthogonal axes (surge [*X*]; anterior–posterior, sway [*Y*]; lateral and heave [*Z*]; dorsoventral) were separated into two components; gravitational and dynamic acceleration. Gravitational acceleration, representing sensor orientation with respect to the Earth's gravity, was first estimated by applying a 4 s box smoother algorithm to each of the raw acceleration axes [78]. Dynamic acceleration, due to physical movement by the turtle, was then calculated by subtracting static acceleration from each of the raw acceleration axes. ODBA was then determined by summing the absolute values of dynamic acceleration across all three body axes [79,80]:

2.1 $$\mathrm{ODBA}=\;|{\mathrm{DAcc}}_{X}|+|{\mathrm{DAcc}}_{Y}|+|{\mathrm{DAcc}}_{Z}|,$$

where DAcc is the dynamic acceleration for each axis; *X*, *Y* and *Z*. ODBA was resampled to a 1 Hz continuous timeseries and mean ODBA (g) was calculated for each dive phase (table 1).

To characterize the turtles' body angle, pitch in degrees was first calculated from raw acceleration values in IGOR Pro, as per Tuck [81]:

2.2 $$\mathrm{Pitch}=\arctan\left(\;\frac{{\mathrm{Acc}}_{X}}{{\mathrm{Acc}}_{Y^{2}}+\;{\mathrm{Acc}}_{Z^{2}}}\;\;\right)\frac{180}{\pi },$$

where Acc is the raw acceleration for each axis; *X, Y* and *Z*. Since it was impossible to align the tag on the carapace parallel to the anterior–-posterior body axis, pitch was corrected for each individual to account for variation in tag placement angle, by determining the pitch value when the turtle was swimming horizontally at a constant depth and subtracting this value from all pitch values [82]. Pitch angles (°) were resampled to a 1 Hz continuous timeseries and mean pitch was calculated for each dive phase (table 1).

To evaluate the tortuosity of movement paths, we first derived heading, or trajectory with respect to magnetic North. We used the *Dead Reckoning Wizard* in the program Framework4 (http://framework4.swan.ac.uk/; [83]) to estimate heading for each individual, requiring input of the raw accelerometer and magnetometer data in all three orthogonal axes. Heading (resampled to 1 Hz) was converted from degrees to radians using the *R* package *NISTunits* [84]. We then applied the *circ.disp* function from the *R* package *CircStats* [85] to estimate circular variance of heading (*V*) for each dive phase, which we used as a measure of tortuosity [86]. Tortuous movement was represented by values of *V* nearer to 1, while uni-directional, straight path travel or the turtle remaining in place was represented by *V* values nearer to 0.

### 2.4. Data analysis

Preliminary analysis of the dive variables (table 1) considered unsupervised clustering techniques commonly used to analyse time-depth data for sea turtles, akin to classification of dive types [24,87–89]. To do this, we selected two commonly used unsupervised clustering algorithms, k-means and hierarchical clustering. Both clustering methods have enabled classification of dive types from time-depth data for a range of diving animals, including sea turtles [24,87–90], and aim to partition *n* observations (dives) into *k* clusters, from which cluster characteristics can then be attributed to inferred behaviour [91]. After combining the dive variables (table 1) for all individuals, the resultant data matrix was standardized (centred and scaled by variable to mean 0 and s.d. 1) to avoid giving variables with larger magnitudes greater importance. We tested 1–10 clusters (*k* = 1–10) for both *k*-means and hierarchical clustering methods using base *R* functions *kmeans* and *hclust*. We did not increase *k* beyond 10, because this negates any benefit sought from clustering into distinct groups of similar-type dives [89].

We then tested for multicollinearity in the dive variables, because this has potential to complicate any inferences of behaviour and hinder interpretation of subsequent statistical analyses [92]. A correlation matrix of standardized diving behaviour variables revealed significant multicollinearity between them (*p* > 0.05) (electronic supplementary material, figure S3). As such, we used principal component analysis (PCA) to objectively transform the multicollinear dive variables into a reduced set of new uncorrelated features characterizing dives. We implemented PCA on the correlation matrix of the original dive variables (standardized to avoid distortion by the differing scales of variables) using the *prin.comp* function in base R [75]. Following PCA, there were 16 principal components (PCs), where for each PC, each dive had a single score to which each of the original dive variables contributed. We retained a subset of the PCs, by selecting the PCs where variance (expressed by the unscaled eigenvalue) was greater than 1 (latent root criterion) [88,93]. These retained PCs were treated as the new features characterizing diving [94], hereafter referred to as multivariate dive features.

To interpret each multivariate dive feature (according to the PC scores), we examined their eigenvector loadings using a cut-off value of ±0.4 [95,96] (electronic supplementary material, figure S7). Since subsequent statistical analysis relied on our interpretations of these multivariate dive features, we ensured that our interpretations were not sample dependent. To do this, we first compared the absolute loadings from the original PCA to mean absolute loadings (±95% confidence intervals) from PCA performed on 1000 bootstrapped samples (with replacement; electronic supplementary material, figure S4). For intuitive interpretation and to aid visualization of final models (see figure 5), if the strongest magnitude loading for a PC was negative, all loadings for that PC were multiplied by −1 and all scores for the PC were recalculated.

### 2.5. Statistical analysis

To determine the seasonal, diel and tidal effects on the diving behaviour of flatback turtles during the foraging life-history stage, we used generalized additive mixed models (GAMMs) [97]. Notably, the GAMMs allowed us to test whether there was a biologically meaningful signal contained in the identified multivariate dive features. We implemented one GAMM for each of the multivariate dive features, using a continuous timeseries of the PC scores for each multivariate dive feature as the dependent response variable. Prior to fitting models, scores were log transformed if required to meet model assumptions [98], then fitted using a Gaussian distribution and identity link error structure. Explanatory variables season, time of day (hour), tide height (m) and tidal strength and direction (m) were calculated for each dive from its mid-point date and time (table 2). Season was selected as a categorical variable representing seasonal water temperature (*T_w_*), rather than treating *T_w_* recorded by the data-loggers as a continuous variable due to disparity between sampling seasons (Summer $\bar{x}\pm s.d.=31.9\pm 0.6^{\circ }C$, Winter $\bar{x}\pm s.d.=22.1\pm 0.7^{\circ }C$). Tide height was calculated from hourly sea level using data collected from local tide gauge observations (table 2; Australian Bureau of Meteorology, 2020). We then calculated tide strength and direction from the hourly change in tide height, representing the magnitude of water movement during ebb (−) or flood (+) tides. Time of day (hour) was treated as a circular variable using a cyclic smoother (*bs* = ‘cc') in the GAMMs and Turtle ID was included as a fixed random effect in each model, to avoid pseudo-replication and account for individual differences in diving behaviour.

**Table 2.** Explanatory variables used for generalized additive mixed models to determine seasonal, diel and tidal effects on the diving behaviour of flatback turtles at Yawuru Nagulagun Roebuck Bay, Western Australia. Variables calculated from the mid-point date and time for each dive. Units of measurement and data source are included.

explanatory variable	type	unit	data source
season	categorical	n.a.	^a^
time of day	continuous	hour	^a^
tide height	continuous	metre	^b^
tide diff (strength and direction)	continuous	metre	^b^

^a^CATS-multi-sensor tag programmed to AWST (GMT + 8).

^b^Hourly Sea-level data, Tide Gauge # 1159, Broome, Western Australia (http://www.bom.gov.au/oceanography/projects/abslmp/data/index.shtml; Australian Bureau of Meteorology).

All possible combinations of categorical and continuous explanatory variables were fitted, including categorical variable interactions, using the R packages *FSSgam* (v. 1.11) [99] and *mgcv* (v. 1.8-31) [100]. Final models were selected according to ranked Akaike's information criterion corrected for small sample size (AICc) [97], which is equivalent to AIC at large sample sizes [101]. If a model was within two AICc units of the model with the lowest AICc value, the model with the fewest variables was selected to avoid over-fitting (electronic supplementary material, table S1). To account for serial-dependence between dives for each individual turtle, correlation structure was added to each of the model fits [102]. Using the auto-correlation function in the *nlme* package (v. 3.1-149) [103], we found a decline of auto-correlation with increasing lag and added the correlation at lag = 1 as a fixed term (*corAR1*) to each final model [97] (electronic supplementary material, table S1). Finally, for each final model, we predicted the effects of the explanatory variables on diving behaviour using *evaluate.smooth* or *evaluate.parametric.term* functions within the *gratia* package (v. 0.4.1) [104].

## 3. Results

**Figure 1.**
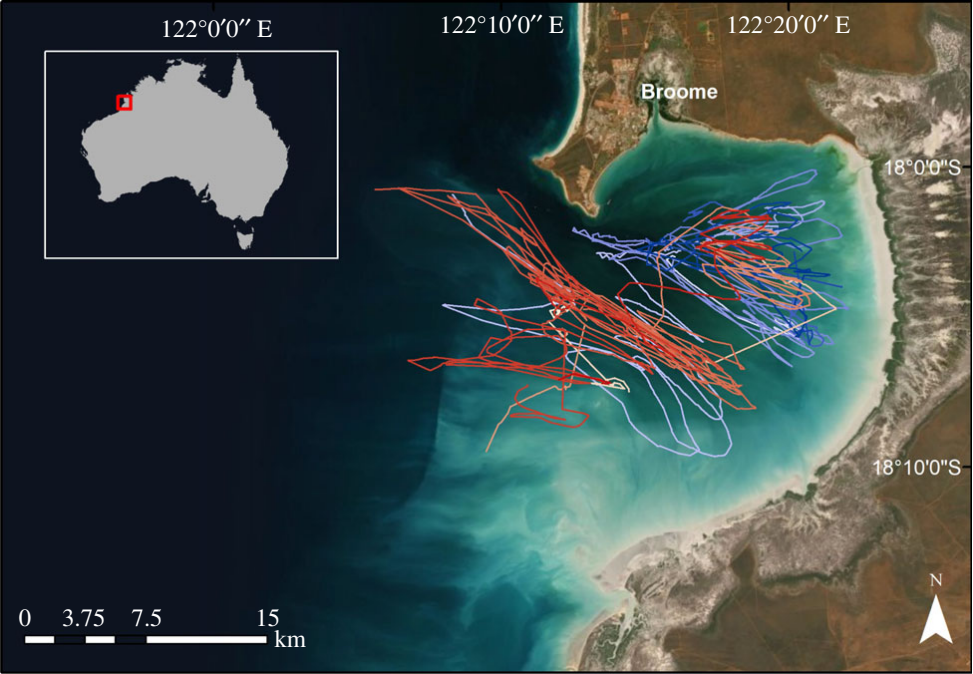
Surface movement paths from filtered GPS locations (CRS: WGS 84) for flatback turtles (*n* = 24) at Yawuru Nagulagun Roebuck Bay, Western Australia, visualized using ArcMap (v. 10.6). For each individual, GPS locations were filtered based on number of satellites (greater than 6.0) and altitude (0–150.0 m) to reduce potentially erroneous locations. Track colours indicate sampling season: Austral summer, warm; winter, cool.

**Figure 2.**
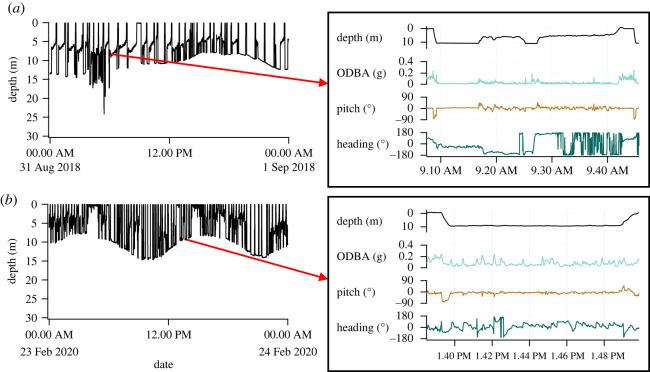
Representative timeseries of flatback turtle diving during (*a*) winter (Turtle ID 9) and (*b*) summer (Turtle ID 31). Inset panel shows concurrent depth, overall dynamic body acceleration (ODBA; proxy for locomotory activity), pitch (body angle) and heading (travel path direction with respect to North) calculated from the multi-sensor data, from which dive variables were derived (table 1 for full explanation of variables).

From 2018 to 2020, we successfully recovered tags from all 28 flatback turtles. Data from four individuals were discarded due to incomplete records (e.g. missing depth data). From the remaining 24 individuals, 11 during Austral summer and 13 in winter, we analysed a total of 56.1 days of multi-sensor data (electronic supplementary material, table S2). During this time, all individuals displayed apparently linear surface movements and remained within Roebuck Bay (figure 1). Turtles ranged in size from 72.5 to 98.9 cm CCL ($\bar{x}=86.6\pm 4.8\;\mathrm{cm}$, *n* = 23) and in mass from 46.0 to 97.0 kg ($\bar{x}=74.4\pm 10.7\;\mathrm{kg}$, *n* = 18) (electronic supplementary material, table S2). In total, we identified 4128 individual dives (table 3), with individual turtles showing diverse diving patterns (figure 2).

**Table 3.** Summary statistics for flatback turtle dives (*n* = 4128 dives) during Austral summer and winter at Yawuru Nagulagun Roebuck Bay, Western Australia, 2018 – 2020. PDSI = post-dive surface interval, bold text represents $\bar{x}\pm s.d.$ per season and overall.

season	Turtle ID	*n* dives	dive frequency (dives/ day)	mean dive depth (m)	maximum dive depth (m)	mean dive duration (min)	maximum dive duration (min)	mean PDSI duration (min)	maximum PDSI duration (min)
winter	6	51	24.5	10.4 ± 3.5	19.3	50.5 ± 15.7	75.9	8.2 ± 15.7	32.5
	7	194	62.2	8.5 ± 5.7	39.4	13.0 ± 13.8	101.3	10.1 ± 13.8	170.8
	8	172	72.6	7.9 ± 3.4	28.0	18.0 ± 18.5	79.3	1.8 ± 18.5	6.1
	9	193	55.0	12.6 ± 10.9	84.5	22.5 ± 14.1	57.7	3.7 ± 14.1	25.9
	10	131	42.3	13.9 ± 15.8	86.1	24.6 ± 14.2	89.2	9.6 ± 14.2	97.3
	11	24	30.4	17.0 ± 7.8	31.9	32.5 ± 14.2	55.5	4.9 ± 14.2	7.8
	13	36	45.6	20.4 ± 15.9	57.2	27.5 ± 16.1	69.7	4.2 ± 16.1	11.3
	15	105	106.1	8.2 ± 2.3	16.7	11.0 ± 9.6	51.8	2.2 ± 9.6	10.1
	17	14	28.6	12.1 ± 1.1	14.0	44.4 ± 45.4	112.2	6.1 ± 45.4	36.3
	18	100	113.6	8.5 ± 12.0	66.6	10.5 ± 11.6	58.8	1.8 ± 11.6	10.0
	19	163	173.4	5.3 ± 1.5	8.3	5.8 ± 5.8	40.7	2.4 ± 5.8	21.3
	20	182	61.9	8.6 ± 5.7	54.8	18.1 ± 19.5	111.5	4.8 ± 19.5	34.1
	22	159	52.5	11.1 ± 13.4	72.9	25.3 ± 16.7	75.0	2.2 ± 16.7	11.4
			**66.8 ± 42.0**	**11.1** ± **4.1**	**44.6 ± 27.4**	**23.3** ± **13.2**	**75.3 ± 22.9**	**4.9** ± **2.9**	**36.5 ± 47.0**
summer	23	60	77.9	11.7 ± 2.9	15.4	16.7 ± 10.5	37.5	1.9 ± 10.5	4.4
	24	82	132.3	8.1 ± 2.2	11.5	9.7 ± 5.4	21.9	1.3 ± 5.4	4.2
	25	180	464.0	9.7 ± 2.1	13.9	2.5 ± 1.3	9.2	0.5 ± 1.3	1.9
	26	37	107.2	10.8 ± 2.8	12.8	11.6 ± 4.7	20.5	1.8 ± 4.7	4.0
	27	128	195.7	7.9 ± 1.7	10.5	6.2 ± 3.6	19.7	1.1 ± 3.6	3.3
	28	392	67.5	12.1 ± 4.5	52.9	19.2 ± 8.6	43.4	2.1 ± 8.9	12.2
	29	430	77.0	11.4 ± 3.1	18.5	16.5 ± 6.4	40.2	2.2 ± 6.4	20.0
	30	349	55.5	11.2 ± 4.6	19.8	19.3 ± 8.9	38.5	6.5 ± 8.8	80.6
	31	593	90.0	10.3 ± 6.7	64.9	11.9 ± 8.0	39.9	3.9 ± 8.0	73.6
	32	248	71.1	13.5 ± 3.5	22.2	17.4 ± 8.2	37.1	2.8 ± 8.2	12.2
	33	105	213.5	7.9 ± 2.9	12.3	4.9 ± 2.9	12.7	1.8 ± 2.9	5.8
			**141.0 ± 118.7**	**10.4** ± **1.9**	**23.1 ± 18.2**	**12.4** ± **6.0**	**29.1 ± 12.4**	**2.7** ± **1.6**	**20.2 ± 28.7**
TOTAL	24	4128	**100.8 ± 92.0**	**10.8** ± **3.2**	**34.8 ± 25.6**	**18.3** ± **11.7**	**54.3 ± 29.9**	**3.7** ± **2.7**	**29.1 ± 39.7**

### 3.1. Developing an analytical technique to interpret diving behaviour

#### 3.1.1. Classification of dive types using unsupervised clustering techniques

**Figure 3.**
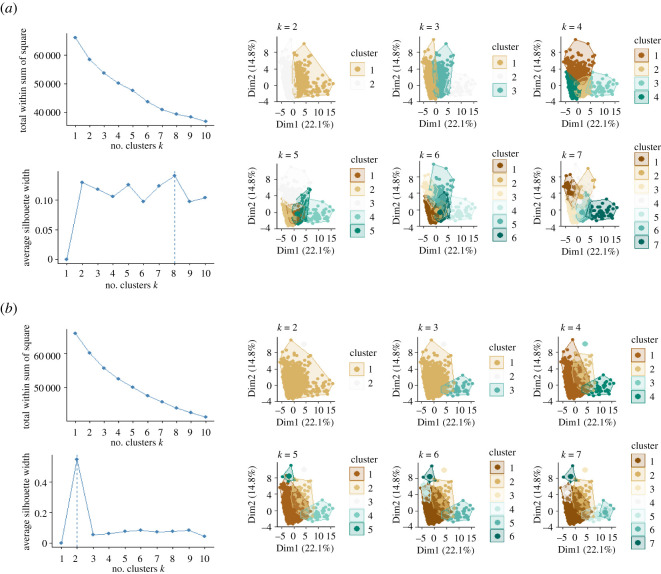
Cluster analysis output for (*a*) *k*-means and (*b*) hierarchical clustering. Elbow and silhouette plots (*k* = 1 : 10) and cluster plots (*k* = 2 : 7) were assessed for each method, to determine optimal number of clusters for *n*_dives_ = 4128 from standardized dive variables (refer table 1), derived from multi-sensor data collected from adult flatback turtles (*n* = 24).

Neither *k*-means nor hierarchical cluster analysis were successful, with both techniques failing to identify distinct clusters, or dive types, from the kinematic and three-dimensional dive variables derived from multi-sensor data. We were unable to determine the appropriate number of clusters (*k*) from the elbow plot of within-cluster sum of squares for both methods (*k*-means, figure 3*a* and hierarchical, figure 3*b*). While the maximum average silhouette width indicated the optimal number of clusters was 8 (*k*-means, figure 3*a*) or 2 (hierarchical, figure 3*b*), cluster plots revealed significant overlap and lack of partitioning between clusters when *k* > 2 for both methods (figure 3).

#### 3.1.2. Characterizing dives from multivariate dive features

PCA determined that the first five PCs (PC1–PC5) had eigenvalues greater than 1.0 (electronic supplementary material, figure S5), therefore these PCs were retained as new uncorrelated multivariate dive features representative of flatback turtle diving behaviour. The total variance explained by the retained PCs (PC1–PC5) was 64.8%; 22.1%, 14.8%, 11.3%, 10.3% and 6.3%, respectively. There was seasonal variance in diving behaviour represented by the principal components (electronic supplementary material, figure S6).

The effect of each of the original dive variables on the multivariate dive features varied in strength and direction, according to the loading contributions meeting the threshold of ±0.4 (electronic supplementary material, figure S7). However, according to the loading contributions, the multivariate dive features (PC1–PC5) each primarily aligned with one specific dive phase (electronic supplementary material, figure S7).

**Figure 4.**
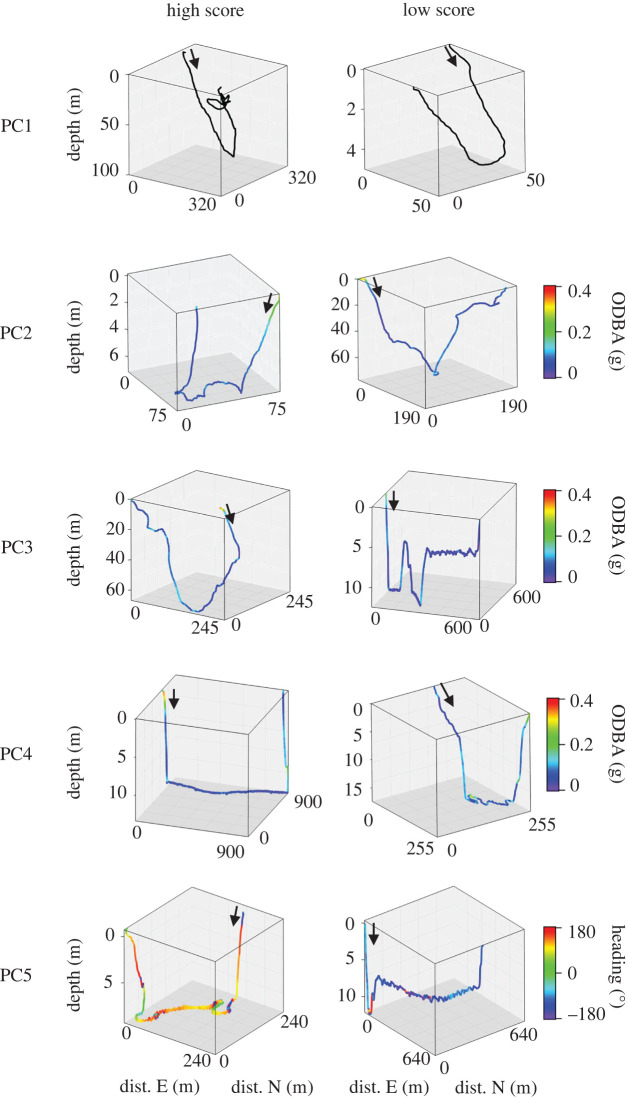
Representative dives for multivariate dive features for flatback turtles. Reconstructed three-dimensional dives representative of high (left column) and low (right column) value scores for each of the retained principal components, PC1–PC5. Black arrow marks dive path start and travel direction, path colour scale for each PC as per interpreted eigenvector loading values, where PC1 = depth, PC2 = activity and body angle (descent), PC3 = activity and body angle (ascent), PC4 = activity and duration (bottom) and PC5 = tortuosity (ascent) (see electronic supplementary material, figure S7). Cartesian coordinates (distance eastwards and northwards from a starting point of 0) were estimated from dead reckoned accelerometer and magnetometer data (20–50 Hz) using the dead reckoning wizard within Framework4 [83]; Vectorial Dynamic Body Acceleration (VeDBA) was selected as a proxy for locomotory speed and path anchored to begin at the turtles' capture location. Dive paths were visualized in three-dimensional space by plotting the combined Cartesian coordinates with the corresponding depth data. Note, all representative dives were selected from Turtle ID 9 and axes scales vary between dives to aid visualization.

*PC1* represented increasing *dive depth* (electronic supplementary material, figure S7). Dives with a high PC1 score were deeper and dives with a low PC1 score were shallower (figure 4).

*PC2* characterized the *descent* phase, specifically increasing *activity level* and negative *body angle* (electronic supplementary material, figure S7). Dives with a high PC2 score were typically characterized by more active, steep descent phases (figure 4). Dives with a low PC2 score had a more gradual, less active descent phase (figure 4).

*PC3* characterized the *ascent* phase, specifically increasing *activity level* and positive *body angle* (electronic supplementary material, figure S7). Dives with a high PC3 score were generally characterized by a more active, steep ascent phase (figure 4). Dives with a low PC3 score had a more gradual, less active ascent phase (figure 4).

*PC4* characterized the *bottom* phase, specifically increasing *activity level* and decreasing *duration* (electronic supplementary material, figure S7). Dives with high PC4 scores were characterized by more active bottom phases that were of shorter duration, whereas dives with low PC4 scores had less active, longer duration bottom phases (figure 4).

*PC5* also characterized the *ascent* phase, representing increasing *tortuosity* (electronic supplementary material, figure S7). Dives with high scores for PC5 were characterized by multi-directional, tortuous travel paths during ascent. Dives with low PC5 scores were associated with straight ascents (figure 4).

### 3.2. Environmental determinants of diving behaviour

**Figure 5.**
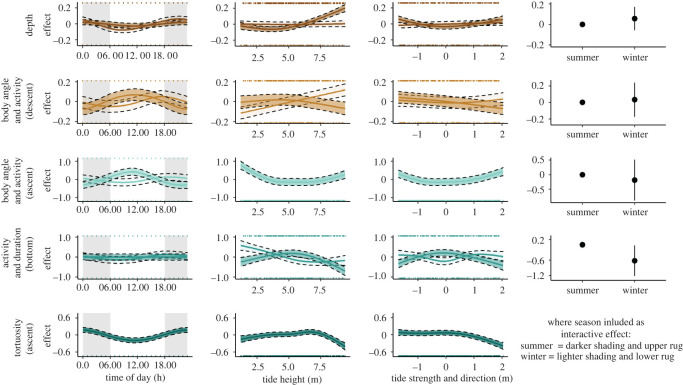
Generalized additive mixed models of the diving behaviour of flatback turtles (*n* = 24) at Yawuru Nagulagun Roebuck Bay, Western Australia. Solid line represents the most parsimonious model with individual specified as a random effect (±s.e. represented by shaded bands and dashed lines; summer, darker shading; winter, lighter shading; table 4). There were clear seasonal, diel and tidal patterns in diving behaviour. Note, to aid visualization the *Y*-axes scales differ between models and for parametric effects. Model colour corresponds to interpretation of PCs by their eigenvector loadings (see electronic supplementary material, figure S7 and figure 4).

There was a significant seasonal effect on the diving behaviour of flatback turtles (table 3, table 4, and figure 5). While the seasonal effect on model intercept was small, there were significant seasonal interactions observed within diel and tidal effects (table 4 and figure 5).

**Table 4.** Summary statistics for the final GAMMs describing the seasonal, diel and tidal effects on the diving behaviour of flatback turtles at Yawuru Nagulagun Roebuck Bay, Western Australia. GAMMS were fitted separately to the scores for each multivariate dive feature (principal components retained from PCA, interpreted by their eigenvector loadings; see electronic supplementary material, figure S7). Model fits shown in figure 5. Each model included Turtle ID as a random fixed effect and a term accounting for auto-correlation structure between dives. All models assumed Gaussian distribution with identity link error structure. Hour = cyclic cubic spline.

model	model formula	d.f.^a^	DE^b^ (%)	DE^c^ (%)
PC1: Depth	∼ s(Hour × Season) + s(Tide.diff × Season) + s(Tide.Height × Season) + Season	15	98.5	98.2
PC2: Body Angle and Activity (descent)	∼ s(Hour × Season) + s(Tide.diff × Season) + s(Tide.Height × Season) + Season	15	97.1	95.7
PC3: Body Angle and Activity (ascent)	∼ s(Tide.diff) + s(Tide.Height) + s(Hour × Season) + Season	11	34.4	6.5
PC4: Activity and Duration (bottom)	∼ s(Hour × Season) + s(Tide.diff × Season) + s(Tide.Height × Season) + Season	15	24.0	5.8
PC5: Tortuosity (ascent)	∼ s(Hour) + s(Tide.diff) + s(Tide.Height)	9	16.8	4.0

^a^Degrees of freedom estimated by the *mgcv* package (v. 1.8–33) implemented in R (v. 4.0.3).

^b^Deviance explained by full model (fixed and random effects).

^c^Deviance explained by full model (fixed effects only).

During summer, turtles dived shallower ($\bar{x}=23.1\pm 18.2\;m$), for shorter durations ($\bar{x}=12.4\pm 5.9\;\min$) and more frequently ($\bar{x}=140.9\pm 118.7\;\mathrm{dives}\;d^{-1}$), with shorter duration post-dive surface intervals ($\bar{x}=2.4\pm 1.6\;\min$) (table 3). While there was a significant diel effect on dive depth (table 4), during summer this effect was negligible (figure 5). However, turtles dived with more active and steeper descent and ascent phases during the day, peaking between approximately 10.00 and 12.00 (figure 5). While turtles displayed no diel pattern for bottom phase activity and duration during summer, there was a tidal pattern (figure 5). Turtles were more active during shorter duration bottom phases when tide height was approximately 5 m and tidal movement was low (figure 5). Conversely turtles were less active during longer duration bottom phases when tidal movement was high, on both ebb and flood tides and when tide height was both low (less than 2.5 m) and high (greater than 7.5 m) (figure 5).

During winter, turtles dived deeper (x=44.6±27.4 m), for longer durations ($\bar{x}=23.34\pm 13.16\;\min$), and less frequently ($\bar{x}=66.8\pm 42.0\;\mathrm{dives}\;d^{-1}$), with longer duration post-dive surface intervals ($\bar{x}=4.8\pm 2.9\;\min$) (table 3). Turtles dived shallower around dawn and deeper after sunset (approx. 19.00; figure 5). The diel pattern observed in winter was similar to summer; turtles dived with more active and steeper descents and ascents during the day (figure 5). However, during winter the peak occurred later in the day (nearer dusk; approx. 17.00–18.00). This coincided with the time that bottom phases were most active and of shorter duration (approx. 18.00; figure 5), therefore turtles were most active in all dive phases (descent, bottom and ascent) at this time. There was an effect of tide height and strength on all aspects of diving behaviour in winter, with the only exception being dive depth (figure 5). In contrast to summer, during winter turtles were more active during shorter duration bottom phases when water movement was high on both ebb and flood tides, and when tide height was low (less than 2.5 m) (figure 5). Turtles performed dives with more active and steeper ascent phases under these tidal conditions.

Regardless of season, turtles dived with straighter ascent phases during the day and on high (greater than 7.5 m) and flood tides, and more tortuous ascent phases at night (figure 5). In general, turtles performed shallow dives (69.2 % maximum depth less than 12.0 m; table 3). The longest recorded dive duration was 112.2 min (Turtle 17), and the maximum recorded dive depth was 86.1 m (Turtle 10), both during winter (table 3).

## 4. Discussion

The main goal of this study was to use multi-sensor biologging data to provide the first detailed description of the environmental influences on the diving behaviour of flatback turtles during their foraging life-history stage. To do so, we objectively condensed a complex multi-sensor dataset into meaningful features and described the characteristics of flatback turtle dives, in detail that would not have been possible with two-dimensional time-depth data alone. Consequently, we were able to provide novel insights into the behaviour of a poorly understood species of sea turtle during its foraging life-history stage. Our approach is likely not only applicable to flatback turtles, but also other species of sea turtle, and those species that can be described as surfacers. Subsequently, our analytical method has the potential to further our understanding of the ecology of a range of species.

### 4.1. Developing analytical techniques to interpret diving behaviour from multi-sensor data

Previous studies have suggested that functional grouping of sea turtle dives could be enhanced with additional data [24]. Therefore, in this study we initially anticipated that the extra kinematic and three-dimensional dive variables derived from multi-sensor tags may have helped reveal distinct dive types from which behavioural function could have been inferred (*sensu* [90,91]). We examined this possibility by considering two multivariate statistical analysis techniques commonly used to classify time-depth data into dive types (e.g. dive profile classification). Both *k*-means and hierarchical clustering methods failed to find discrete underlying structure in the data, and we were unable to group dives into types. While it is possible to subjectively choose the appropriate number of clusters (corresponding to dive type and inferred behavioural function) *a priori*, we observed a high degree of similarity (i.e. overlap) between clusters when using more than two clusters, which would represent an oversimplification of the diverse behaviour of sea turtles [51]. On the other hand, any increase beyond 10 clusters contravenes the original purpose of grouping dives into different types [89]. Combined with the cluster overlap observed, this would have made it difficult to interpret the functions corresponding to those dive types.

The absence of dive types was not surprising, considering that traditional techniques to analyse diving behaviour were originally developed for time-depth data collected mainly from endothermic divers (and not surfacers). As ectotherms, sea turtles have lower metabolic rates resulting in longer dive durations than endothermic divers [105]. As a result of these longer dive durations, multiple behaviours are exhibited during one dive meaning specific dive function cannot always be generalized from typical dive types, for example foraging and resting during U-shaped dives [19,23,24]. The addition of kinematic and three-dimensional dive variables derived from multi-sensor tags enabled us to better capture the complexity of this behavioural diversity. Subsequently, this additional information actually decreased the likelihood of any dives having attributes similar enough to group together as ‘dive types'. This is consistent with more recent studies using novel behavioural parameters such as activity and angular velocity, who found that classic dive types do not correspond directly to distinct behaviours [106,107], further confirming that sea turtles perform multi-purpose dives.

We present a viable alternative method for analysing diving behaviour from multi-sensor biologging data. Although clustering was unsuccessful, this did not impede our ability to interpret our data in a biologically meaningful manner for our study species. PCA allowed us to first condense this complex dataset objectively into meaningful multivariate features, to then infer the diving behaviour of flatback turtles. PCA is a popular tool used in ecological and behavioural studies to analyse multi-dimensional, complex datasets such as ours [94,95,108] and effectively eliminated the multicollinearity typical of all diving behaviour data [94]. This multicollinearity likely concealed any underlying structure in the original dive variables [109]. PCA revealed interpretable structure in the multivariate dive features (principal components), and in turn highlighted some of the complexities in the diverse diving behaviour of surfacers. For instance, when activity loaded strongly and positively onto a principal component, this coincided with dive duration loading negatively (see electronic supplementary material, figure S7), reflecting the relationship between di-oxygen consumption and aerobic dive limits (ADL) [42,110]. The collinearity between dive variables may be an important contributor to the ineffectiveness of clustering techniques.

### 4.2. Environmental drivers of flatback turtle diving behaviour during the foraging life-stage

The success of our approach is evident in the biologically meaningful signal contained in the multivariate dive features in relation to environmental variation. Our PCA-GAMM method allowed us to describe the seasonal, diel and tidal effects on flatback turtle diving behaviour as a result of the additional information from multiple sensors, instead of subjectively accepting any number of multicollinear dive variables or poorly defined dive types. Overall, the significant seasonal difference observed in diving behaviour was expected, given that water temperature at the study site varied by approximately 10°C between Austral summer and winter. As ectotherms, a sea turtle's physiological rates are strongly temperature dependent, resulting in greater di-oxygen consumption at warmer temperatures, with flow on effects of decreased ADLs [42,67,111,112]. The reduced ADL, coupled with greater energy demands that accompany warmer temperatures, in turn requires turtles to alter aspects of their diving behaviour. Here, for flatback turtles, this includes reduced dive durations and increased dive frequencies to ensure continued energy gain alike foraging green turtles (*Chelonia mydas*) [113,114].

#### 4.2.1. Diel patterns in diving behaviour

Depth, which was the primary parameter represented in the first principal component, is a fundamental aspect of behaviour for any diving animal; in particular for benthic foragers like the flatback turtle, as it reflects both physiological limits and behavioural choice through habitat selection. For example, in green and hawksbill turtles (*Eretmochelys imbricata),* shallower and shorter dives during the day are alleged to be the result of spatio-temporal changes in behaviour, where foraging occurs in shallow waters and deeper waters are selected for nocturnal resting [90,114–116]. Here a similar diel pattern in dive depth was observed during winter, with shallower day-time dives and deeper night-time dives, but not during summer. While the maximum recorded dive depth in this study (approx. 86 m) was well within previously recorded maxima for the species, most dives were shallow (less than 12 m) and probably limited by the uniformly shallow bathymetry of the nearshore study site (generally less than 20 m, except for one deep channel on the northwestern edge of the bay) [52,53,55].

In addition to dive depth, we also found a diel pattern in principal components 2, 3 and 5 which are related to characteristics of descents and ascents. During the day, flatback turtles dived with both steeper and more active descents and steeper, straighter, more active ascents in comparison to dives performed at night. Such steeper dives are expected to reduce the time taken for turtles to commute between the surface and depth, ultimately increasing foraging time at the seafloor. Equally, steeper, more active descents and ascents will also ensure that turtles broadly remain within the same foraging patch. Like other sea turtle species, flatback turtles are probably visual foragers, requiring daylight to identify and locate potential prey items [117,118]. Therefore, in accordance with optimal foraging theory [119], this diurnally increased diving activity is expected to maximize benthic foraging opportunities, the principal foraging strategy of the flatback turtle [62,120]. Conversely, at night ascent phases were less active, more tortuous, with less steep body angles signifying less directed movement between the seafloor and the surface. Prolonged ascents in particular have been widely documented in sea turtles, where the initial ascent from the seafloor is interrupted at the depth of neutral buoyancy and the turtle proceeds to spend an extended time at this depth, presumably for energy efficient mid-water transport in the absence of buoyant forces [19,52,53,110,121–123].

Considering the strong diel patterns in descent and ascent behaviour, it was surprising that we did not find any appreciable diel pattern in the locomotory activity of turtles during the bottom phase of dives, given high activity has been considered proxy for foraging behaviour in other taxa [124]. This may have been an artefact of functionally very different behaviours being characterized by similar raw ODBA values. While feeding on sessile benthic prey and resting on the seafloor, the turtle is stationary with a horizontal body position (J Hounslow *et al.,* personal observation, 2018–2021). As such, the kinematics of these behaviours may have been indistinguishable from each other in original dive variables. For other species of sea turtle, resting was identified (in part) from variance of body posture, determined by gravitational acceleration, rather than lack of locomotory activity determined by dynamic accelerations (i.e. ODBA) [125]. This issue may be specific to carnivorous flatback turtles, since foraging was well detected from posture-related parameters for herbivorous green turtles, who grazed with upwards and downwards body angles instead of horizontal [125]. Although our data suggest periods when flatback turtles are foraging or resting, these behaviours may have been better detected specifically in the bottom phase of dives using alternative multi-sensor derived variables or a head mounted device to capture subtle head movements, rather than a carapace-mounted device (cf. [126]).

Alternatively, considering the role of light in mediating predator–prey interactions, it may be that the diurnal patterns in diving behaviour described here are not determined by the need to feed, but the need to avoid being eaten [127]. Both tiger sharks (*Galeocerdo cuvier*) and saltwater crocodiles (*Crocodylus porosus*) prefer shallow habitats overlapping with the flatback turtles' near shore range and are encountered in Yawuru Nagulagun Roebuck Bay [128–131]. Predation risk for sea turtles is highest while commuting between the surface and seafloor, where they are presumably most visible and easily ambushed [132]. Therefore, the steeper and more active descents and ascents during daylight hours may reduce exposure to visual predators such as tiger sharks [133]. This risk-reduction strategy may be crucial in an environment where turtles face high predation risk [61]. Meanwhile, night dives characterized by more tortuous, less active, gradual ascents for mid-water transport could be attributed to resting periods while predation risk is reduced under the cover of darkness [52,134].

While diel patterns were evident in all aspects of diving behaviour quantified here, we discovered that the timing of those patterns differed seasonally. The vespertine activity during winter, instead of diurnal activity during summer, might be a behavioural consequence of the ectothermic physiology of turtles and their temperature-dependant metabolic rates. Sea turtles can surface-bask for thermoregulatory heat-uptake by solar radiation to aid a number of physiological processes, such as digestion (cf. [135,136,137]). While thermoregulation might not be necessary in warmer summer waters, cold winter temperatures may render this behaviour beneficial and displace more active diving behaviour to the afternoon rather than the middle of the day [135,138]. Indeed, flatback turtles were more likely to engage in extended post-dive surface intervals in winter, further supporting the conclusion that thermoregulation is an important feature of their behaviour during winter and matching anecdotal observations at Yawuru Nagulagun Roebuck Bay (J. Hounslow, personal observation, 2018–2021). In addition to thermoregulation, the seasonal shift in the timing of activity may be related to seasonal changes in diet and prey distribution [139]. In particular, this seasonal change may represent a shift from benthic foraging in summer to pelagic foraging in winter, potentially arising from flatback turtles following the same diel vertical migrations of gelatinous zooplankton and jellyfish [140,141]. Although flatback turtles feed on a range of benthic soft-bodied invertebrates including sea cucumbers and sea pens [55,60], pelagic foraging has also been reported [55,142] and flatback turtles have been observed gorging on sea tomato jellyfish *Crambione magistophora* during winter (J. Hounslow, personal observation, 2018–2021).

#### 4.2.2. Tidal patterns in diving behaviour

Tide is an important abiotic factor influencing ecological processes within coastal ecosystems, yet few studies have characterized how it influences the diving behaviour of sea turtles (e.g. [114]). At Yawuru Nagulagun Roebuck Bay, tide appeared to have a greater influence on flatback turtle diving behaviour than time of day, which is not surprising considering the study site experiences tidal ranges up to 11 m [143]. Animals inhabiting super-tidal coastal areas are expected to have evolved behavioural strategies to effectively use or alternately, avoid any negative consequences of tidal movement. Overall, we found that flatback turtles altered their diving behaviour particularly when exposed to high tidal flows and extreme tides. For example, the trajectory of ascent phases became increasingly directional on high, flood tides, to potentially exploit the vast intertidal areas of Yawuru Nagulagun Roebuck Bay that become accessible on these tides [71,144]. This is similar to other species of sea turtle that use high tides to access foraging habitat such as mangroves [145] or feed on benthic prey as they emerge upon inundation in the intertidal [114,146]. Conversely, we found similar effects for both ebb and flood tides on the behaviour of turtles during bottom and ascent phases of dives, suggesting that behaviour is somewhat similar between rising and falling tides. Kemps Ridley turtles (*Lepidochelys kempii*) exposed to large tides swam in the opposite direction of tidal currents during both descents and ascents, possibly to reduce displacement while foraging [147]. While flatback turtles appear to be predictably displaced along the presumed linear paths of tidal currents (figure 1z), the increased locomotory effort and steeper dive angles observed during ascent would reduce the time exposed to passive drift in the water column and therefore reduce displacement by tidal flow. Similar to diel patterns, we observed significant seasonal interactions within the effect of tide on the diving behaviour of flatback turtles. This suggests that seasonal changes in either biotic or abiotic conditions mediate how flatback turtles respond to this dynamic environment. The synchronization of behaviour to tidal flows may be linked to different strategies used to exploit tidal currents, such as selective tidal transport or continuous tidal transport [148]. Future analysis should compare the behaviour and orientation of turtles in relation to the direction of tidal flow, to understand the navigational ability of turtles in such a dynamic environment.

## 5. Conclusion

As biologging technologies continually advance, the number of sensors integrated into one device expands, giving us access to increasingly complex datasets. While we cannot directly attribute behavioural function to any data without direct validation, our ability to characterize complex features of diverse diving behaviour is equally improving [33,80]. Alongside rapid uptake of biologging technologies, we highlight that we must also strive to constantly evolve our analysis of such data, and here we demonstrate a viable alternative for analysing diving behaviour from colinear multivariate datasets derived from multi-sensor biologgers. Notably, we provide the most detailed interpretation of diving behaviour for the least-studied species of sea turtle, during a poorly understood life-history stage. Flatback turtles altered their diving behaviour according to their environment (e.g. extreme tides, wide ranging sub-tropical water temperatures), to thermoregulate and avoid predators while optimizing foraging. Future directions should consider techniques that permit fine-scale validation of these multi-sensor biologging data [45,125,126,149].

## Data Availability

The source code and processed data supporting this article have been made available as part of the electronic supplementary material [150].
